# Free cMSAP flap for the treatment of chronic osteomyelitis with complex soft tissue defects in foot and ankle

**DOI:** 10.3389/fcimb.2025.1689817

**Published:** 2025-11-24

**Authors:** Shu Cao, Yongqiang Xu, Sheng Xiao, Zili Xu, Runxin Chen, Xiang Zhang

**Affiliations:** Department of Orthopedics, Hunan Provincial People’s Hospital, The First Affiliated Hospital of Hunan Normal University, Changsha, China

**Keywords:** chronic osteomyelitis, soft-tissue defects, cMSAP flap, foot, ankle

## Abstract

Chronic osteomyelitis is very difficult to treat, numerous reconstructive strategies have been described for repairing soft tissue defect combined with chronic osteomyelitis in the foot and ankle. The free chimeric medial sural artery perforator (cMSAP) flap has been recommended for reconstructing the complicated wounds. We conducted a retrospective study on 16 patients who accepted cMSAP flap to reconstruct the soft tissue defect in the setting of chronic osteomyelitis in foot and ankle from January 2021 to December 2023. The effect of surgery and the functional evaluation of the limb were assessed. Results displayed that no chronic osteomyelitis was recurrent, no cases needed bulky flap defatting and the functions of the lower extremity were all improved at the last follow-up. The free cMSAP flap is a good alternative for reconstructing complex soft tissue defects with deep dead space in the setting of chronic osteomyelitis in the foot and ankle.

## Introduction

Osteomyelitis is an infection of the bone that mainly originates from three sources: hematogenous dissemination, direct inoculation (due to an open fracture or surgery), or adjacent dissemination (due to infection of the adjacent soft tissue) (Lew and Waldvogel, 2004). Chronic osteomyelitis was defined as the presence of bone infection for at least 6 weeks (Park and Kim, 2010). Chronic osteomyelitis is very difficult to treat due to antibiotic resistance, osteonecrosis, sequestrum formation, and other reasons (Lew and Waldvogel, 2004). Recurrence of chronic osteomyelitis appears to be a persistent barrier, leading to 30% therapeutic failure rate (Shannon et al., 1973).

There are three main components for the successful reconstruction of an osteomyelitic limb with complex soft tissue defects: restoration of the bony framework, obliteration of dead space, and restoration of soft-tissue coverage (Hong et al., 2017). That means a single reconstruction procedure requires both restoration of superficial soft-tissue defects and appropriate obliteration of dead space.

In the literature, numerous reconstructive strategies, such as the pedicled fasciocutaneous flap and the myocutaneous flap, have been described to repair soft tissue defect combined with chronic osteomyelitis in the foot and ankle. Pedicled fasciocutaneous flap such as distally based peroneal artery perforator-plus fasciocutaneous flap is appropriate to reconstruct such soft tissue defects, owing to the advantages of the large dimension, survival reliability, and the simple surgical procedure (Luo et al., 2020). However, it is hard to carry the muscle flap to fill the dead space of the fasciocutaneous flap, which may lead to the recurrence of chromic osteomyelitis. Another useful method is myocutaneous flap transplantation because of its advantages such as rich blood supply, a large size for harvesting, and effective anti-infection ability. The shortcoming of the myocutaneous flap is the limited mobility between the skin and muscle as well as an unsatisfactory recipient site appearance (Huang et al., 2003).

In recent years, the chimeric perforator flap, named as the perforator flap, in combination with different blocks of tissue supplied by one source vessel, having greater spatial freedom between skin flap and muscle flap, more flexible design, and superior aesthetic outcomes, has been recommended for the reconstruction of complicated wounds with deep dead space (Sui et al., 2023). The free chimeric medial sural artery perforator (cMSAP) flap not only can carries part of the medial gastrocnemius muscle to embed in the deep dead space but also can restore the skin defect in a single reconstructive procedure. However, there are few reports on cMSAP flap to treat chronic osteomyelitis in the foot and ankle. The purpose of this article is to validate the efficacy of cMSAP flap to reconstruct the soft tissue defect of chronic osteomyelitis in the foot and ankle based on a retrospective review of 16 patients with a minimum follow-up period of 1 year.

## Patients and methods

This retrospective study enrolled patients with a diagnosis of chronic osteomyelitis in the foot and ankle who underwent surgical reconstruction with cMSAP flap at our hospital between January 2021 and December 2023. This study was conducted in accordance with the Declaration of Helsinki and received approval from the Ethics Committee of Hunan Provincial People’s Hospital (no. 2025-184). Written informed consent was acquired from all patient parents and/or their legal guardians. A total of 16 patients (13 male and three female) participated, with an average age of 49.2 (range, 14–72) years. The clinical data of these patients are listed in **Table 1**. Wound secretion bacterial culture and drug sensitivity tests prior to the surgery were performed; sensitive antibiotics were used according to the results. Preoperative X-ray and MRI were performed among all patients to evaluate the pathology of chronic osteomyelitis. Preoperative computed tomography angiography (CTA) of the lower extremities was carried out to assess the quality of the recipient vessels, and color Doppler ultrasonography was applied to locate the perforators of the medial sural artery in the donor site (Thione et al., 2004; Tao et al., 2024).

**Table 1:** Characteristics of the patients.

Case	Gender	Age	Duration (months)	Site of chronic osteo-myelitis	Cause	Size of skin flap (cm)	Size of muscle flap (cm)	Recipient vessels	Donor site	Follow-up months
1	M	15	2	Tibia	Traffic injury	8 × 5	4 × 3	PTV	Primary closure	24
2	M	57	12	Fibula	Traffic injury	10 × 5	5 × 3	ATV	Primary closure	30
3	M	53	24	Tibia	Crushing injury	10 × 5	5 × 3	PTV	Primary closure	36
4	F	66	6	Calcaneus	Traffic injury	12 × 5	7 × 3	PTV	Primary closure	40
5	F	14	12	First metatarsal	Postoperative infection	9.5 × 3.5	5 × 3	DPV	Primary closure	18
6	M	46	2	Second metatarsal	Crushing injury	8 × 6	5 × 4	DPV	Primary closure	24
7	M	72	2	Tibia	Traffic injury	12 × 6	7 × 3	PTV	Primary closure	18
8	F	69	2	Tibia	Traffic injury	12 × 6	6 × 5	PTV	Primary closure	36
9	M	49	2	Second metatarsal	Crushing injury	9 × 5	5 × 3	DPV	Primary closure	24
10	M	54	2	Tibia	Crushing injury	8 × 5	5 × 4	PTV	Primary closure	12
11	M	16	4	Tibia	Postoperative infection	15 × 6	10 × 3	PTV	Primary closure	12
12	M	66	24	Second metatarsal	Crushing injury	14 × 6	6 × 3	DPV	Primary closure	12
13	M	60	2	Tibia	Postoperative infection	17 × 6	8 × 4	PTV	Primary closure	12
14	M	43	2	Tibia	Traffic injury	14 × 6	6×4	PTV	Primary closure	12
15	M	73	2	Tibia	Traffic injury	10 × 6	6×3	PTV	Primary closure	12
16	M	34	2	Tibia	Crushing injury	15 × 6	8 × 6	PTV	Primary closure	12

PTV, posterior tibia vessels; ATV, anterior tibia vessels; DPV, dorsalis pedis vessels.

### Operative technique

Radical debridement of the infected bone and soft tissues is the important guarantee of a successful construction surgery. For chronic osteomyelitis in Cinerny and Mader (C-M) stages III and IV, trepanation and partial or intraosseous bone resection were performed. The inflammatory cancellous bone and tissue in the medullary cavity were carefully debrided, and the local infected soft tissue was removed without any leniency. After thorough debridement, the wound was covered with vacuum sealing drainage (VSD) and continuously irrigated with sterile normal saline for a week (Chang et al., 2025).

Reconstruction surgery was carried out when the wound irrigation bacterial culture test was negative. The patients were placed in the supine position with the hip abducted and externally rotated and the knee flexed at 90°. Tourniquet was routinely used on the thigh. A paper template was created according to the dimensions of the soft tissue defect in the foot and ankle. Based on the perforators marked with the preoperative color Doppler ultrasonography and the axis of the cMASP flap (the line from the popliteal crease midpoint to the medial malleolus midpoint), the design of the skin paddle was outlined on the main perforator as the center, and the size of the skin paddle was approximately 1 cm larger than the paper template in periphery. The muscle paddle was designed according to the bone dead space area feature (Thione et al., 2004). Firstly, the anteromedial border of the flap was incised (Lin et al., 2025), and then the main perforator during dissection in the superficial fascia was identified. The perforator was retraced back to the main trunk of the medial sural artery through the “retrograde four-side dissection technique” (Tang et al., 2013). The medial gastrocnemius muscle paddle nourished by the independent branches of the medial sural artery was harvested to an appropriate size according to the volume of the bone dead space. The main trunk of the medial sural vessel proximal to the bifurcation was dissected (Lin et al., 2021). After harvesting the cMSAP flap, the muscle paddle was inserted into the bone dead space, and the skin paddle was fixed in the skin defects in the recipient site. The medial sural vessels were anastomosed to the proper recipient vessels, avoiding pedicle compression or kinking. The donor site of the flap was closed directly.

### Efficiency assessment

The postoperative clinical evaluations of the patients were performed as outpatients every 3 months after the reconstruction surgery for the first year; then, a 6-month outpatient visit was requested. The American Orthopaedic Foot and Ankle Society (AOFAS) scoring system was used to assess foot and ankle joint function, and two-point discrimination testing was used to evaluate the sensory recovery of the flap (Bell-Krotoski et al., 1993). A visual analog scale (VAS) score was performed to assess the cosmetic appearance of the flap. A score of 0 indicated the worst cosmetic outcome and 10 indicated the best cosmetic outcome.

### Statistical analysis

Data was analyzed using SPSS statistical analysis software program version 28.0 (SPSS Inc., Chicago, IL, USA). Continuous variables were summarized as means and standard deviations (SD). Student’s *t* test was used to compare paired data. Statistical significance was considered at *p ≤*0.05.

### Case report

**Figure 1:**
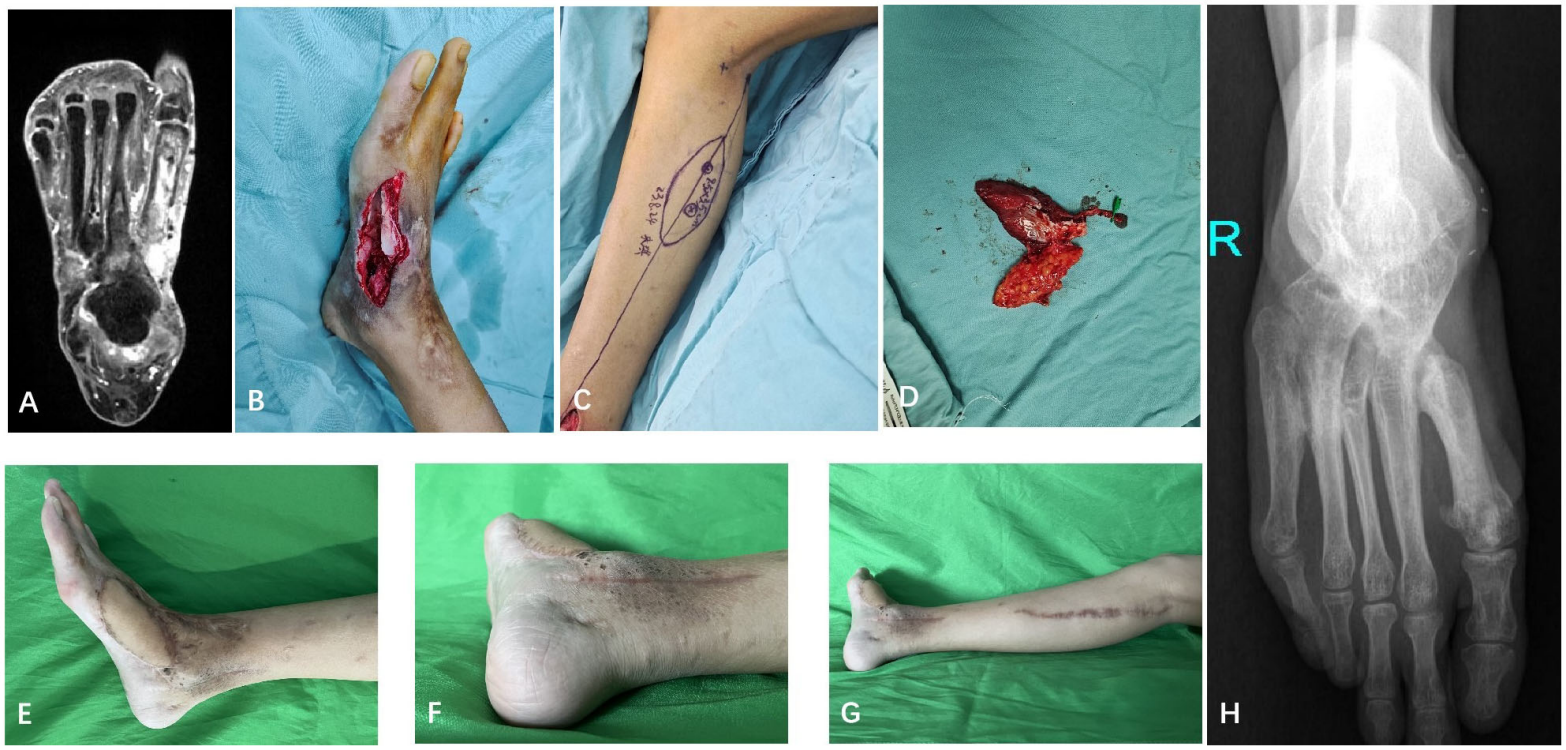
A 14 years old girl (case 5) suffered a chronic osteomyelitis in the first metatarsal. **(A)** Preoperative MRI manifested the diffuse infection lesion of the first metatarsal and around soft tissue, which belonged to Cinerny and Mader type IV. **(B)** The trepanation and partial infected bone resection were performed, the inflammatory soft tissue was debrided. A moderate-sized skin defect and a dead space were left after radical debridement. **(C, D)** A cMASP flap was designed and harvested. **(E-G)** Postoperative view of the donor and recipient site at 18 month follow-up. **(H)** The X-ray of the last follow-up.

**Figure 2:**
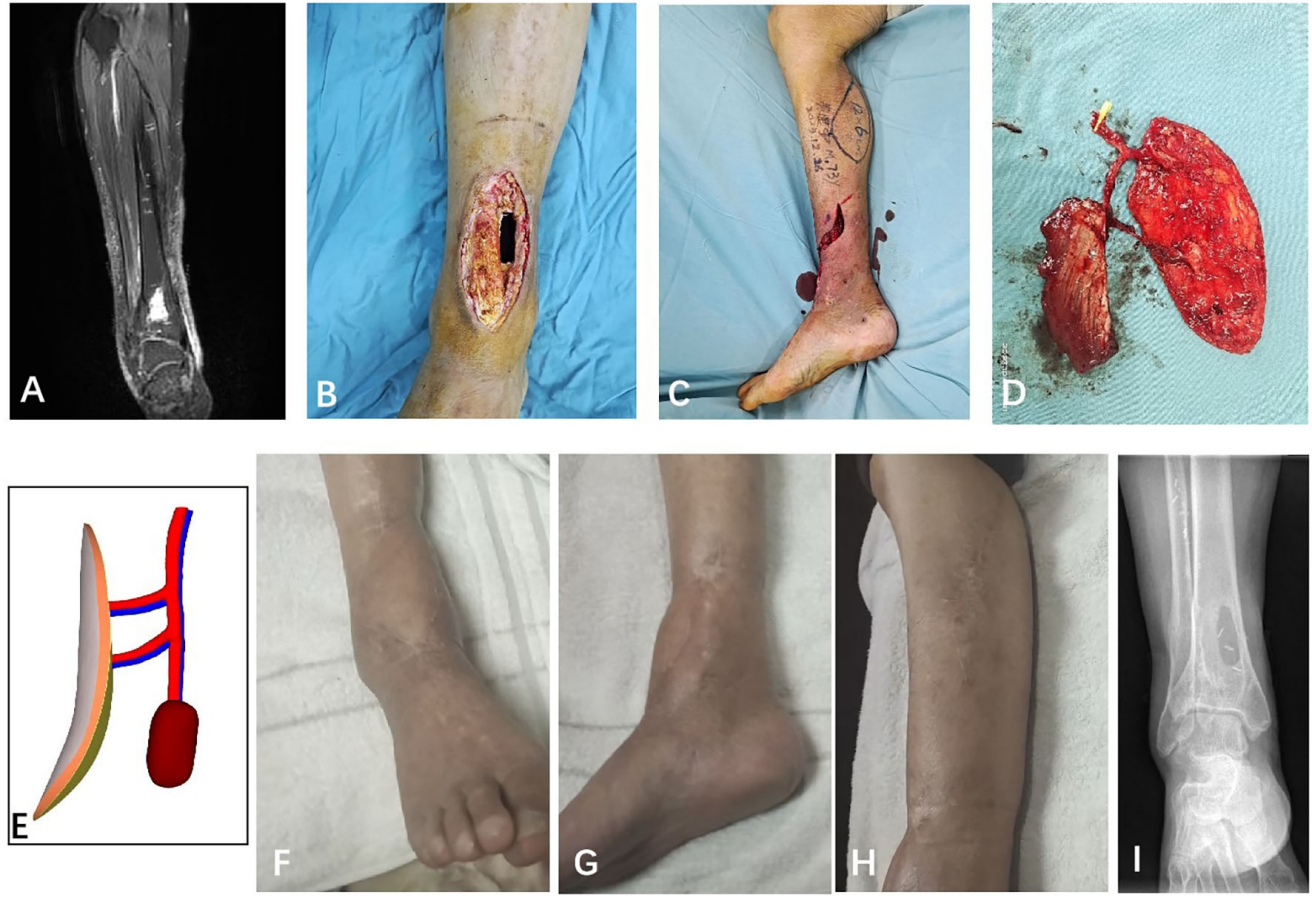
A 72 years old man (case 7) suffered a chronic osteomyelitis in the distal of the tibia after a open fracture. **(A)** Preoperative MRI manifested the localized infection lesion of the tibia and around soft tissue, which belonged to Cinerny and Mader type III. **(B)** The trepanation and partial infected bone resection were performed, the inflammatory soft tissue was debrided. **(C, D)** A cMASP flap was designed and harvested. **(E)** The diagram shows the muscle paddle is nourished by the main trunk of the medial sural artery (MSA). **(F-H)** Postoperative view of the donor and recipient site at 12 month follow-up. **(I)** The X-ray of the last follow-up.

A case report is shown in **Figures 1** and **2**.

## Results

All of the chronic osteomyelitis originated from direct inoculation, including 13 cases from open fractures and three from previous surgery. Preoperative bacterial culture results revealed primarily methicillin-resistant *Staphylococcus aureus* (11 cases); vancomycin was used as the antibiotic of choice in these instances. The size of the skin paddle of the cMSAP flap ranged from 8 cm × 5 cm to 17 cm × 6 cm, and the size of the medial sural muscle flap ranged from 3 cm × 3 cm to 8 cm × 5 cm. All of the cMSAP flaps survived uneventfully.

The mean period of follow-up was 20.9 (range, 12–36) months. At the last follow-up, no chronic osteomyelitis was recurrent in the cases. The AOFAS scores were significantly improved compared with the preoperative scores (**Table 2**). According to the two-point discrimination testing results, all of the patients regained protective sensation. The mean VAS score for the aesthetic appearance of the flap was 8.5 (**Table 2**). No cases needed bulky flap defatting, and the affected foot can wear the same size shoes of the contralateral foot in all the cases.

**Table 2:** Preoperative and last follow-up AOFAS scores, two-point discrimination testing, and VAS results.

Items	AOFAS scores (mean ± SD)	Two-point discrimination testing (mm)	VAS score
Preoperative	59.9 ± 6.23		
Last follow-up	95.6 ± 2.42	21.0 ± 3.72	8.5 ± 0.73
*t*	-18.14		
*P*-value^a^	0.000		

a *t* test; *P* ≤0.05, statistically significant.

## Discussion

Chronic osteomyelitis, characterized by infected dead bone and local vascular insufficiency within a compromised soft tissue envelope, presents significant challenges for both physicians and patients because of its high recurrence rates (Lew and Waldvogel, 2004). Adequate debridement must be managed to prevent recurrence, which often leads to a large dead space of the affected bone and soft tissue defects needing reconstruction procedures. The myocutaneous flap is the best means of fighting a recurrent infection due to the advantage of providing vascular supply to both the muscle and the overlying skin (Lew and Waldvogel, 2004). The free myocutaneous flaps are reliable workhorses for the treatment of chronic osteomyelitis in the foot and ankle where there is no appropriate muscle. The muscle paddle can eliminate the dead space of the affected bone, and the skin paddle can cover the soft tissue defects, wherein only one group of blood vessels need to be anastomosed for free flaps during the operation, thus reducing the operation time and risk. However, the free myocutaneous flaps are limited by the bulky appearance and great damage of the donor site.

Recently, with the introduction and popularity of perforator flaps, which can provide a large skin area, long vascular pedicle, and customized thickness and volume, the superiority of the myocutaneous flap has been challenged (Kao et al., 2009a). Besides that, perforator flaps can be used in combination with different blocks of tissue supplied by different branches of vessels with a common pedicle as a chimeric flap (Hallock, 2008; Hallock, 2008).

The skin and subcutaneous adipose tissue located in the foot and ankle are thin. Reconstruction of soft tissue with dead space in this region requires an ultrathin perforator flap to allow for a normal fit into footwear (Qing et al., 2018). The free chimeric medial sural artery perforator flap provides a good solution to reconstruct soft tissue defects with deep dead space in the foot and ankle because the skin of the posterior upper calf region is uniformly thin, and the flap does not need to be microdissected thin during the reconstruction procedure, thus saving operation time. Meanwhile, the result of the flap appearance in our study was satisfactory; no cases need debulking.

Compared with the anterolateral thigh perforator (ALTP) flap with a chimeric vastus lateralis muscle for the reconstruction of the soft tissue defects with deep dead space in the foot and ankle, the flap thickness of the ALTP flap was significantly thicker than the MSAP flap in both sexes on ultrasonographic studies (Haq et al., 2025). The thickness of both flaps had an appositive correlation with BMI. The relationship was stronger for ALTP flap in male and MSAP flap in female (Doan et al., 2018). Microdissected debulking of the ALTP flap primarily or the thin ALTP flap secondarily at least 12 months post-operatively often was performed for a satisfactory appearance (Yang et al., 2006), and the ALTP flap was recommended for large defects of the foot due to the large-sized flap and long pedicle (El-Gammal et al., 2013). In our study, none of our patients necessitated to have thinning of the MSAP flap in the foot and ankle since aggressive primary thinning can result in flap compromise or subject the patient to a second debulking operation.

With regard to the free radial forearm flap, which was about half of the thickness of the MSAP flap (Arman Danielian et al., 2023; Haq et al., 2025), the MSAP flap has presented less donor site morbidity (Zhang et al., 2025). It can help avoid sacrificing the radial artery, which is one of the two major blood vessels supplying the upper limb, and skin grafting for direct closure at the donor site with a width of 7 cm (Kao et al., 2009b). Regarding comparisons of MSAP and conventional gastrocnemius myocutaneous flaps, MSAP flaps can only provide the skin paddle, and the conventional gastrocnemius myocutaneous flap has less freedom for inset. Conventional medial or lateral gastrocnemius muscle or musculocutaneous flaps have drawbacks of bulkiness and donor site morbidity (Lasserre et al., 2011). From our point of view, the MSAP flap is a good alternative flap for foot and ankle defect coverage.

Elimination of the dead space of the infected bone with the muscle paddle of the cMSAP flap was essential, and failure to eliminate the dead space often results in necrosis of vascular flaps due to hematoma and the recurrence of chronic osteomyelitis (Qing et al., 2023). The muscle paddle of the cMSAP flap was classified into three types by Sui (Sui et al., 2023) according to the nourished branches or vessels of the skin and muscle paddles, which clearly defined the advantage, disadvantage, and indication of each type. A working algorithm had been introduced for the reconstruction procedure according to this classification (Qing et al., 2023), which can be facilitated for the reconstructive surgeons and trainees to gain a better understanding of chimeric flap designs and allow aesthetically superior reconstruction of the complex defects. In our study, most of the muscle paddle classifications are type I. In addition, we found another type of flap for the larger soft tissue defects: the two perforator vessels of the MSA were dissected to nourish the large skin paddle whose area exceeds the maximum size of one perforasome, and the muscle paddle was nourished by the main trunk of the MSA with abundant blood and oxygen supply and can effectively fight infections. Meanwhile, the vascular pedicle of the muscle paddle was sufficiently long to be freely rotated up to 180° to avoid pedicle kinking and twisting (**Figure 2E**).

Otherwise, the medial sural cutaneous nerve can be carried in the flap to restore the sensation in the flap (Pan et al., 2017). Although we did not reconstruct the sensory nerve of the cMSAP flap during the procedure in the study, the flaps usually regained their protective sensory innervations from the recipient bed at 1 year after operation.

In our last follow-up, the AOFAS scores showed that all of the patients had excellent or good walking ability. No one complained of muscular weakness or fatigue while climbing and walking down stairs.

Based on our previous clinical work experience, when applying this flap, several points should be noted. First, preoperative Doppler ultrasound and CTA should be used to locate the perforators of the medial sural artery and evaluate the vascular conditions of the recipient site; this routine preoperative work can reduce the risk of flap-related complications. Second, the diameter of the medial sural artery always was smaller than its accompanying medial sural vein, so dissection of the main trunk of the medial sural artery and vein before bifurcation allows easy microanastomosis with the recipient vessels (Lin et al., 2021). Otherwise, the risk of flap failure due to late-onset venous thrombosis seems to be somewhat higher than the more established free flaps because of the mismatched dimensions for the medial sural vein and recipient vessels (Alharbi et al., 2023; Coulet et al., 2025). In our study, a microvascular stapler was always used for vein anastomosis, and no vascular compromise was detected in our patients.

The limitations of our study include its retrospective design, the small number of patients, and heterogeneous etiology of the defects.

## Conclusion

The free cMSAP flap is a good alternative for reconstructing complex soft tissue defects with deep dead space in the setting of chronic osteomyelitis in the foot and ankle. The skin paddle can cover the skin surface defects, and the muscle paddle can fill the dead space against infection. This flap has lower donor site morbidity and offers a good reconstructive and aesthetic result without the need for debulking in the future.

## Data Availability

The original contributions presented in the study are included in the article/supplementary material. Further inquiries can be directed to the corresponding author.
